# Mode of delivery and cord blood cytokines: a birth cohort study

**DOI:** 10.1186/1476-7961-4-13

**Published:** 2006-09-26

**Authors:** Ngoc P Ly, Begoña Ruiz-Pérez, Andrew B Onderdonk, Arthur O Tzianabos, Augusto A Litonjua, Catherine Liang, Daniel Laskey, Mary L Delaney, Andrea M DuBois, Hara Levy, Diane R Gold, Louise M Ryan, Scott T Weiss, Juan C Celedón

**Affiliations:** 1 Channing Laboratory, Department of Medicine, Brigham and Women's Hospital and Harvard Medical School, Boston, MA, USA; 2 Division of Pediatric Pulmonary Medicine, Department of Pediatrics, Massachusetts General Hospital for Children and Harvard Medical School, Boston, MA, USA; 3 Division of Pulmonary and Critical Care Medicine, Department of Medicine, Beth Israel Deaconess Medical Center, Boston, MA, USA; 4 Department of Biostatistics, Harvard School of Public Health, Boston, MA, USA; 5 Division of Pediatric Pulmonary Medicine, Children's Hospital of Boston and Harvard Medical School, Boston, MA, USA

## Abstract

**Background:**

The mechanisms for the association between birth by cesarean section and atopy and asthma are largely unknown.

**Objective:**

To examine whether cesarean section results in neonatal secretion of cytokines that are associated with increased risk of atopy and/or asthma in childhood. To examine whether the association between mode of delivery and neonatal immune responses is explained by exposure to the maternal gut flora (a marker of the vaginal flora).

**Methods:**

CBMCs were isolated from 37 neonates at delivery, and secretion of IL-13, IFN-γ, and IL-10 (at baseline and after stimulation with antigens [dust mite and cat dander allergens, phytohemagglutinin, and lipopolysaccharide]) was quantified by ELISA. Total and specific microbes were quantified in maternal stool. The relation between mode of delivery and cord blood cytokines was examined by linear regression. The relation between maternal stool microbes and cord blood cytokines was examined by Spearman's correlation coefficients.

**Results:**

Cesarean section was associated with increased levels of IL-13 and IFN-γ. In multivariate analyses, cesarean section was associated with an increment of 79.4 pg/ml in secretion of IL-13 by CBMCs after stimulation with dust mite allergen (P < 0.001). Among children born by vaginal delivery, gram-positive anaerobes and total anaerobes in maternal stool were positively correlated with levels of IL-10, and gram-negative aerobic bacteria in maternal stool were negatively correlated with levels of IL-13 and IFN-γ.

**Conclusion:**

Cesarean section is associated with increased levels of IL-13 and IFN-γ, perhaps because of lack of labor and/or reduced exposure to specific microbes (e.g., gram-positive anaerobes) at birth.

## Background

According to the Centers for Disease Control and Prevention's National Center for Health Statistics, the rate of cesarean section rose to 29.1% in 2004 in the United States (a > 40% rate increase since 1996) [1]. Because cesarean section has been associated with increased risks of asthma [2-5] and atopy [6-8], further understanding of the relationship between mode of delivery and immune system ontogeny is needed.

Several studies have shown immunological differences between children with and without atopy at the time of birth. For example, increased cord blood levels of IL-13 have been positively associated with atopy among children with a family history of atopy [9-11]. Although less consistent, increased cord blood levels of IFN-γ have been associated with atopy among children with a family history of atopy [11,12]. In children at risk for atopy, increased neonatal levels of IL-10 have been associated with reduced risk of egg allergy[10] but increased risk of atopic dermatitis [11,12]. Among children unselected for family history, detectable neonatal IL-10 was associated with a reduced risk of asthma at age 6 years[13].

We hypothesized that mode of delivery influences neonatal immune responses. Specifically, we examined whether cesarean section results in neonatal secretion of cytokines associated with increased risk of atopy and/or asthma in childhood. We were also interested in exploring potential mechanisms for any observed association between mode of delivery and neonatal immune responses.

In murine models [14], oral exposure to lipopolysaccharide (LPS) during passage through the birth canal triggers gut epithelial cell activation, as measured by production of the chemokine MIP-2 and activation of the transcription factor NF-κB. In contrast, activation of gut epithelial cells does not occur in mice delivered by cesarean section. These findings suggest that microbial exposure during passage through the birth canal may trigger immune responses leading to tolerance in mice.

During the natural birthing process of humans, neonates transition from the sterile environment of the womb to a nonsterile environment where they are exposed to microbes originating from their mother and the surrounding environment. Neonates born by vaginal delivery acquire most of their intestinal flora by swallowing their mother's vaginal fluid at birth. In contrast, children born by cesarean section are not exposed to the maternal vaginal flora at birth. We examined whether specific microbes in the maternal intestinal flora (which is closely correlated with the maternal vaginal flora) [15-18] has different influences on neonatal immune responses depending on mode of delivery.

## Methods

### Study cohort

Pregnant women were recruited between July 2003 and November 2005 from three outpatient facilities affiliated with Brigham and Women's Hospital in Boston at their 24-week prenatal visit. Inclusion criteria were maternal age between 18 years and 44 years; plans to deliver at Brigham and Women's Hospital; and maternal ability to speak English or Spanish. Informed consent was obtained from participating mothers. The study was approved by the Institutional Review Board of the Brigham and Women's Hospital.

### Questionnaire and review of medical records

A questionnaire was administered to each participating woman between her 24-week prenatal visit and delivery to obtain information on demographics, general health, and history of allergic diseases and/or symptoms in herself and the father of her child. Information on labor and delivery (including the white blood cell count of participating mothers) was obtained from review of medical records.

### Isolation of Cord Blood Mononuclear Cells

Cord blood samples were collected by needle/syringe from the placental side of the umbilical vein after the newborn was delivered but prior to placental delivery. Samples were processed within 24 hours, and cord blood mononuclear cells (CBMCs) were isolated from umbilical cord blood by density gradient centrifugation with Histopaque (Sigma-Aldrich, St. Louis, MO).

### Cytokine measurements

Aliquots of 4 × 10^5 ^CBMCs were incubated in triplicate in 96-well round-bottom tissue-culture plates (Corning, Acton, MA) at 37°C in 5% CO_2_. At the start of the culture, cells were either unstimulated (media) or stimulated with each of the following antigens: dust mite allergen (*Der f *1) at 30 μg/ml, cat dander allergen (*Fel d *1) at 10 μg/ml (Indoor Biotechnologies, Charlottesville, VA), phytohemagglutinin (PHA) at 10 μg/ml, and LPS at10 μg/ml. Cell supernatant fluids were harvested 24 hours after stimulation and analyzed in duplicate for cytokine (IL-13, IFN-γ, IL-10) production by ELISA according to the manufacturer's instructions (Pierce Biotechnology, Inc., Rockford, IL). The sensitivities of the assays were < 2 pg/ml for IFN-γ, < 7 pg/ml for IL-13, and < 3 pg/ml for IL-10.

### Stool collection and culture

A stool sample was collected from participating women between their 24-week prenatal visit and delivery. More than a gram of stool was collected into a sterile specimen container and frozen for transport to the laboratory. Samples were weighed, serially diluted (10^-2 ^to 10^-7^) with sterile phosphate-buffered-saline (PBS) in an anaerobic chamber, and plated onto enriched or selective agar media. Media for recovery of obligate anaerobes included pre-reduced Brucella base blood agar containing 5% sheep blood, hemin, and menadione (BMB, PML, Tuluatin, OR), and BMB containing 5% laked sheep blood, 100 mcg/ml of kanamycin, and 7.5 mcg/ml vancomycin (BKV) for *Bacteroides *and *Prevotella *species. Facultative organisms were isolated with tryptic soy base 5% sheep blood agar (BAP, PML, Tuluatin, OR), bile esculin azide agar for *Enterococci*, MacConkey's agar for *Enterobacteriaceae*, and Rogosa selective agar for lactobacilli and bifidobacteria.

Following incubation under appropriate atmospheric conditions and lengths of time, as recommended for the recovery of the specific groups of microorganisms, colonies were enumerated on the various selective media, and individual colony types were selected for identification by gram stain, based on colony morphology. All counts were recorded as log_10 _CFU/gram dry weight sample. The lower limit of detection of the various organisms was 1.5 log_10 _CFU/gram.

### Statistical analysis

The distribution of cytokine levels was skewed, with a significant number of undetectable values; therefore, median cytokine levels are presented. Differences in the levels of cord blood cytokines between neonates born by vaginal delivery and those born by cesarean section were examined by nonparametric two-sample Wilcoxon tests. In complementary analyses adjusting for potential confounders, we estimated the effect of mode of delivery on cytokine secretion by CBMCs by stepwise linear regression. In these analyses, cytokine values were log_10_-transformed. In addition, we estimated the odds of having detectable cytokine levels at birth for children born by cesarean section compared with those born by vaginal delivery using stepwise logistic regression. In the final models, we included variables that were significant at P < 0.05 or that satisfied a change in estimate criterion (≥ 10%) in the parameter estimate.

The following variables were considered for inclusion in the multivariate analysis: race/ethnicity, gender, gestational age, birth weight, birth length, Apgar score, maternal age, and maternal history of atopy (a physician's diagnosis of any of the following: asthma, eczema, hay fever, or allergy).

In exploratory analyses, we examined whether the maternal gut flora (a close correlate of the maternal vaginal flora) had different influences on neonatal cytokine production depending on mode of delivery. We calculated Spearman's correlation coefficients (r_s_) for the number of microbes for specific bacterial species in maternal stool and cytokine levels in cord blood, first in all subjects and then after stratification by mode of delivery (vaginal vs. cesarean section). All analyses were performed with SAS version 8 (SAS Institute, Cary, NC).

## Results

### Population characteristics

Table 1 shows the characteristics of the 37 participating mother-child pairs. The mean age of participating women was 33.3 years (standard deviation [SD] = 6.1 years); approximately 60% of participating women had a history of atopy. Of the 37 participating children, 22 (59.5%) were born by vaginal delivery. Of the 15 women who had a cesarean section, (66.7%) had an elective cesarean. Only one study participant used probiotics during pregnancy. Maternal infection and/or use of antibiotics were not exclusion criteria in our study. The mean neonatal gestational age was within normal limits. One neonate had an Apgar score of 1 at 5 minutes, was intubated, and admitted to the neonatal intensive care unit (NICU); this neonate was delivered by non-elective cesarean at 37 weeks of gestation. Of note, exclusion of this child did not appreciably change the results of our analyses; thus, results are presented for all subjects. There were no significant differences in race/ethnicity, maternal age, maternal history of atopy, maternal smoking during pregnancy, neonatal gender, gestational age, birth length, or Apgar score between children born by cesarean section and those born by vaginal delivery. Children born by cesarean section were more likely to weigh more at birth than those born by vaginal delivery.

**Table 1:** Characteristics of Maternal-Infant Pairs in Relation to Mode of Delivery

	**Total (N = 37)**	**Vaginal Delivery (N = 22)**	**Cesarean Section (N = 15)**	**p-value**
	**N (%)**			
Race/Ethnicity				
*White*	25 (67.6)	14 (63.6)	11 (73.3)	
*Black*	3 (8.1)	2 (9.1)	1 (6.7)	
*Hispanic*	8 (21.6)	5 (22.7)	3 (20.0)	
*Others*	1 (2.7)	1 (4.6)	0 (0)	0.83
Gender				
*Male*	21 (56.8)	12 (54.5)	9 (60)	
*Female*	16 (43.2)	10 (45.5)	6 (40)	1.00
Maternal history of atopy*	22 (59.5)	14 (63.6)	8 (53.3)	0.53
Maternal smoking during pregnancy	2 (5.4)	1 (4.6)	1 (6.7)	1.00
NICU admission	1 (2.7)	0 (0)	1 (6.7)	0.41
	**Mean (range)**			
Maternal age, years	33.3 (17.9–43.2)	31.9 (17.9–40.6)	35.3 (25.8–43.2)	0.08
Neonatal birth weight, kg	3.4 (2.2–4.4)	3.3 (2.2–4.3)	3.6 (3.0–4.4)	0.03
Neonatal gestational age, weeks	39.0 (36.0–41.0)	38.9 (36.0–41.0)	39.1 (37.0–41.0)	1.00
Neonatal birth length, cm	49.7 (44.0–53.0)	49.5 (44.0–53.0)	50.0 (46.0–53.0)	0.48
APGAR at 5 minutes	8.7 (1–10)	8.9 (8.0–10.0)	8.4 (1.0–10.0)	0.98

*Atopy = doctor diagnosed asthma, eczema, hay fever, or allergies

### Cytokine secretion by mode of delivery

**Figure 1:**
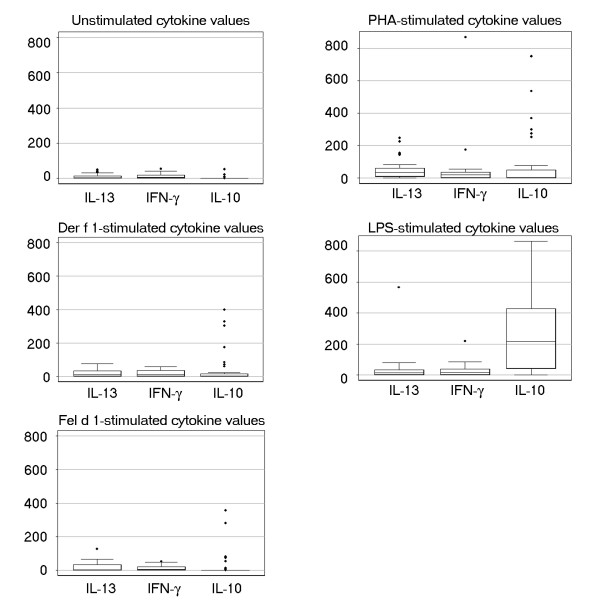
Secretion of IL-13, IFN-γ, and IL-10 cytokines by neonatal CBMCs. IFN-γ, IL-10, and IL-13 secretions were measured in unstimulated supernatants and supernatants after 24 hours' stimulation with allergens (*Der f 1 *and *Fel d 1*), phytohemagglutinin (PHA), and lipopolysaccharide (LPS). The median is represented by the black bar. The upper and lower boundaries of the box represent the 25^th ^to 75^th ^percentiles of the data, respectively. Observations < 1.5 times the height of the box beyond either quartile are displayed within the whiskers. (•) represents outliers.

In Figure 1, we show the distributions of cytokines (IL-13, IL-10, and IFN-γ) produced by neonatal CBMCs at baseline and after stimulation with antigens (*Fel d 1, Der f 1*, PHA, and LPS), which were mostly single-tailed. Whereas IFN-γ had the highest rate of detection by ELISA, IL-10 had the lowest rate of detection, particularly at baseline and after allergen stimulation (Table 2).

**Table 2:** Association between Cytokine Secretion by CBMCs and Mode of Delivery

		**Cytokine Levels (pg/ml)**						
		
		**Vaginal Delivery (n = 22)**			**Cesarean Section (n = 15)**			
		**median**	**ranges**	**% with Detectable Value**^±^	**median**	**ranges**	**% with Detectable Value**^±^	**wilcoxon p-value**^†^
**IL-13**								
	**Media**	0.01*	0.01–47.58	40.9	3.77	0.01–32.96	57.1	0.25
	***Fel d 1***	**0.01**	**0.01–66.27**	33.3	**10.40**	**0.01–126.38**	71.4	**0.04**
	***Der f 1***	**0.01**	**0.01–75.65**	40.9	**26.14**	**0.01–65.61**	85.7	**0.01**
	**PHA**	25.18	0.01–223.54	63.6	39.31	7.50–246.04	100.0	0.22
	**LPS**	5.46	0.01–565.55	50.0	20.19	0.01–44.65	78.6	0.39
**IFN-γ**								
	**Media**	3.69	0.01–53.02	50.0	6.32	0.01–39.75	85.7	0.24
	***Fel d 1***	1.24	0.01–52.00	50.0	9.22	0.01–34.18	85.7	0.41
	***Der f 1***	8.10	0.01–58.93	59.1	16.52	0.01–54.95	85.7	0.43
	**PHA**	11.10	0.01–173.58	63.6	23.23	0.01–866.65	85.7	0.38
	**LPS**	20.03	0.01–218.70	68.2	15.21	0.01–85.27	85.7	0.97
**IL-10**								
	**Media**	0.01	0.01–49.96	18.2	0.01	0.01–0.01	0	0.10
	***Fel d 1***	0.01	0.01–355.80	27.3	0.01	0.01–78.57	14.3	0.37
	***Der f 1***	0.01	0.01–399.46	36.4	0.01	0.01–175.30	28.6	0.44
	**PHA**	21.50	0.01–759.45	59.1	0.01	0.01–367.98	42.9	0.23
	**LPS**	181.91	0.01–787.56	95.5	259.11	0.01–863.05	78.6	0.65

*Low value of 0.01 was assigned to undetectable cytokine level.

^± ^Percentage of cord blood samples in which cytokine measured by ELISA have detectable values within each category of mode of delivery.

^† ^For comparison of cord blood cytokines between neonates born by vaginal delivery and those born by cesarean section.

In bivariate analyses (Table 2), secretion of IL-13 by CBMCs in response to *Fel d 1 *and *Der f 1 *was significantly higher in neonates born by cesarean section than in those born by vaginal delivery. Secretion of IL-13 by CBMCs (at baseline and after stimulation with PHA and LPS) and IFN-γ (at baseline and after stimulation with antigens and PHA) was also higher in neonates born by cesarean section than in those born by vaginal delivery, but such differences were not statistically significant. There was a non-significant trend for reduced secretion of IL-10 by CBMCs at baseline in neonates born by cesarean section.

After adjustment for potential confounders in multivariate linear regression analyses (Table 3), birth by cesarean section was significantly associated with increased secretion of IL-13 by CBMCs after stimulation with allergens, PHA, and LPS. In this multivariate analysis, cesarean section was also associated with increased secretion of IFN-γ by CBMCs at baseline and after stimulation with *Fel d 1 *and PHA. In contrast, cesarean section was associated with reduced secretion of IL-10 by CBMCs at baseline (p = 0.08). In these multivariate models, maternal atopy was independently associated with IL-13 secretion by CBMCs at baseline and after stimulation with allergens and PHA, and with IFN-γ secretion by CBMCs at baseline and in response to allergens. There was no association between maternal atopy and neonatal levels of IL-10.

**Table 3:** Association between Cesarean Section and Cytokine Secretion by CBMCs

		**Adjusted**^‡^	
**Outcome**		β*	**p-value**
**IL-13**			
	**Media**	0.9	0.08
	***Fel d 1***	**1.56**	**0.007**
	***Der f 1***	**1.9**	**0.0006**
	**PHA**	**1.82**	**0.002**
	**LPS**	**1.38**	**0.01**
**IFN-γ**			
	**Media**	**1.24**	**0.02**
	***Fel d 1***	**1.23**	**0.01**
	***Der f 1***	0.90	0.07
	**PHA**	**1.27**	**0.03**
	**LPS**	0.76	0.17
**IL-10**			
	**Media**	-0.66	0.08
	***Fel d 1***	-0.51	0.42
	***Der f 1***	-0.36	0.60
	**PHA**	-0.11	0.88
	**LPS**	-0.19	0.70

*Change of one log10 pg/ml cytokine level in children born by cesarean section as compared to children born by vaginal delivery.

‡Foroutcome IL-13, model adjusted for maternal age, maternal atopy, neonatal birth weight, neonatal gestational age, and neonatal birth length.

For outcome IFN-γ, model adjusted for maternal age, maternal atopy, and neonatal birth weight.

For outcome IL-10, model adjusted for maternal age, neonatal gestational age, and neonatal birth weight.

Our results for the logistic regression analyses of the relation between mode of delivery and detectable cytokine secretion in neonates were similar to those of the linear regression analyses shown above. For example, in multivariate logistic regression analyses, cesarean section was associated with increased odds of having detectable levels of IL-13 in cord blood (odds ratio [OR] = 26.0; 95% confidence interval [CI] = 2.0, 336.8) and IFN-γ (OR = 30.8; 95% CI = 1.7, 555.9) after stimulation with *Fel d 1*. Although not statistically significant, cesarean section was associated with reduced odds of having detectable IL-10 secretion after stimulation with *Fel d 1 *(OR = 0.42; 95% CI = 0.06–2.87).

### Maternal gut flora and cord blood cytokines by mode of delivery

We found no significant differences in the numbers of anaerobic or aerobic bacteria in maternal stool between women who delivered by cesarean section and those who delivered vaginally. There was no significant difference in the composition of the maternal gut flora of atopic and nonatopic women (data not shown).

In the analysis including all subjects (n = 37), total anaerobic bacteria in maternal stool were positively correlated with secretion of IL-10 by CBMCs after stimulation with *Fel d 1 *(r_s _= 0.44, p = 0.008) and *Der f 1 *(r_s _= 0.36, p = 0.03) allergens. In addition, gram-positive anaerobes (lactobacilli and bifidobacteria) were associated with increased IL-10 secretion in response to *Der f 1 *(r_s _= 0.37, p = 0.02), and gram-negative anaerobes (*Bacteroides *and *Prevotella*) were associated with increased IL-10 secretion in response to *Fel d 1 *(r_s _= 0.40, p = 0.02).

Table 4 shows the results of the analysis of the relation between the maternal gut flora and cytokine secretion by CBMCs after stratification by mode of delivery. Among children born by vaginal delivery, total anaerobes and gram-positive anaerobes in maternal stool were each associated with increased secretion of IL-10 by CBMCs after stimulation with *Fel d 1 *and *Der f 1*. In contrast, gram-negative aerobes *(Enterobacteriaceae) *in maternal stool were negatively correlated with secretion of IL-13 (after stimulation with *Der f 1*) and IFN-γ (at baseline and after stimulation with antigens [*Fel d 1*, *Der f 1*, and LPS]).

**Table 4:** Correlation between Maternal Stool Bacteria and Cord Blood Cytokine Secretion by Mode of Delivery

		**Vaginal Delivery (n = 22)**		**Cesarean Delivery (n = 15)**	
			
**Maternal Stool Bacteria**	**Stimulant**	**Cytokines**			
		**IL-13**			
		
**Gram-negative aerobes (Enterobacteriae)**					
		**r**_s_*	**p-value**	**r**_s_*	**p-value**

	**Media**	-0.38	0.08	0.52	0.06
	***Fel d 1***	-0.25	0.27	0.39	0.17
	***Der f 1***	**-0.46**	**0.03**	0.25	0.38
	**PHA**	-0.28	0.21	-0.30	0.30
	**LPS**	-0.35	0.11	0.14	0.62

**Gram-negative anaerobes (Bacteroides/Prevotella)**					
		**r**_s_*	**p-value**	**r**_s_*	**p-value**

	**Media**	-0.27	0.22	**0.59**	**0.03**
	***Fel d 1***	-0.22	0.34	**0.70**	**0.006**
	***Der f 1***	-0.40	0.07	0.49	0.07
	**PHA**	-0.29	0.18	-0.36	0.21
	**LPS**	-0.07	0.77	**0.57**	**0.03**

		**IFN-γ**			
		
**Gram-negative aerobes (Enterobacteriae)**					
		**r**_s_*	**p-value**	**r**_s_*	**p-value**

	**Media**	**-0.52**	**0.01**	-0.10	0.73
	***Fel d 1***	**-0.53**	**0.01**	0.04	0.90
	***Der f 1***	**-0.47**	**0.03**	0.04	0.90
	**PHA**	-0.35	0.11	0.14	0.64
	**LPS**	**-0.50**	**0.02**	0.22	0.44

**Gram-negative anaerobes (Bacteroides/Prevotella)**					
		**r**_s_*	**p-value**	**r**_s_*	**p-value**

	**Media**	-0.34	0.13	0.24	0.40
	***Fel d 1***	-0.30	0.17	0.36	0.21
	***Der f 1***	-0.41	0.06	0.45	0.11
	**PHA**	-0.19	0.40	0.49	0.07
	**LPS**	-0.15	0.50	**0.58**	**0.03**

		**IL-10**			
		
**Gram-positive anaerobes (lactobacilli/bifidobacteria)**					
		**r**_s_*	**p-value**	**r**_s_*	**p-value**

	**Media**	0.35	0.11	±	---
	***Fel d 1***	**0.43**	**0.04**	0.15	0.62
	***Der f 1***	**0.45**	**0.03**	0.30	0.30
	**PHA**	-0.02	0.94	0.23	0.44
	**LPS**	-0.03	0.89	0.23	0.42

**Total Anaerobes**					
		**r**_s_*	**p-value**	**r**_s_*	**p-value**

	**Media**	0.26	0.25	±	----
	***Fel d 1***	**0.48**	**0.03**	0.12	0.69
	***Der f 1***	**0.42**	**0.05**	0.08	0.78
	**PHA**	0.25	0.26	-0.22	0.44
	**LPS**	0.26	0.25	-0.22	0.45

*Spearman's correlations.

± Correlation coefficients were not statistically obtainable due to undectectable IL-10 levels in the media samples (unstimulated CBMCs) among subjects in the cesarean section group.

Among children born by cesarean section, gram-negative anaerobes in maternal stool were associated with increased secretion of IL-13 by CBMCs at baseline and after stimulation with *Fel d 1 *and LPS, and with increased secretion of IFN-γ in response to LPS stimulation (Table 4).

## Discussion

To our knowledge, this is the first study to demonstrate an association between cesarean section and increased neonatal secretion of IL-13 and IFN-γ. This finding provides a potential immunologic basis for previous reports of an association between cesarean section and atopy or asthma [2-8], as elevation of IL-13 [9-11] and IFN-γ [11,12] at birth has been associated with asthma and atopy in childhood. In addition, there was a non-statistically significant trend for an inverse association between cesarean section and neonatal levels of IL-10 (a cytokine with inhibitory effects on the secretion of Th1 and Th2 cytokines in vivo) [19-21]. Although this finding should be further assessed in larger studies, it suggests that abnormal stimulation of mechanisms that downregulate both arms of the immune response (e.g., T regulatory cells [Tregs]) may influence the pathogenesis of atopy in children born by cesarean section [22-24].

The observed association between mode of delivery and neonatal immune responses may be explained by absent or reduced labor in children delivered by cesarean section. The process of labor may directly influence neonatal immune responses, thereby influencing cytokine secretion at birth. Although a relationship between labor and neonatal secretion of IL-13, IFN-γ, and IL-10 has not been shown, the stress of labor has been associated with decreased T lymphocytes and CD4+ helper T cells [25,26], and increased neutrophils [27,28], natural killer (NK) cells [26,28], TNF-α [29], and IL-6 [13,29] in cord blood. In contrast, cesarean section without labor has been associated with increased T lymphocytes and CD4+ helper T cells [25,26], decreased neutrophils [27,28], natural killer (NK) cells [26,28], TNF-α [29], and IL-6 [13,29] in neonates at birth.

Although labor itself may have important immunoregulatory effects on neonates [25-28] and thus partly explain our findings, it is also plausible that the observed neonatal cytokine profile in children born by cesarean section is due to their reduced contact with the maternal vaginal flora at birth. We measured the composition of the maternal gut flora, which is strongly correlated with that of the maternal vaginal flora.

Our findings with regard to bacterial species in the maternal intestinal flora and neonatal immune responses should be interpreted with caution because of small sample size and inability to control for confounders such as maternal diet. However, our preliminary results in children born by vaginal delivery are interesting and suggest the possibility that exposure to specific microbes in the maternal vaginal flora during passage through the birth canal influences neonatal immune responses. In particular, we found that gram-positive anaerobes and total anaerobes in maternal stool were associated with increased secretion of IL-10 by CBMCs, and that gram-negative anaerobes and gram-negative aerobes in maternal stool were associated with reduced secretion of IL-13 and IFN-γ by CBMCs.

The observed association between anaerobes in maternal stool and increased neonatal secretion of IL-10 by CBMCs (at 24 hours after stimulation with allergens) is consistent with results of experiments in murine models and in vitro studies in humans. Stimulation of cord blood lymphocytes with gram-negative bacteria for 24 hours (including anaerobes such as *Bacteroides *species) results in strong secretion of IL-10 [30]. In rodents, peritoneal cells produce IL-10 after stimulation with the CPC of *Bacteroides fragilis *[31]. More specifically, PSA from *B. fragilis *elicits IL-10 production from a population of murine Tregs beginning at 24 hours after stimulation [32]. In murine models, the PSA molecule of *B. fragilis *is presented to T cells by intestinal dendritic cells (DCs) residing at mucosal surfaces, which then activate CD4+T cells and elicit appropriate cytokine secretion resulting in a balanced Th1/Th2 immune response [33]. Moreover, DCs have been shown to mediate the secretion and activity of Tregs [34,35].

Administration of lactobacilli to atopic children has been associated with increased production of cytokines produced by Tregs (e.g., IL-10) [36,37]. It is thus plausible that early modulation of immune responses by specific bacteria (e.g., anaerobes) during passage of the neonate through the birth canal [38] results in upregulation of neonatal Tregs and/or direct downregulation of Th1 and Th2 immune responses. The effects of labor may further interact with those of the maternal gut flora on neonatal immune system development.

The observed association between gram-negative anaerobes and increased secretion of IL-13 (at baseline and after stimulation with *Fel d 1 *and LPS) and IFN-γ (after stimulation with LPS) by CBMCs in children born by cesarean section is intriguing and unexplained. Although it may be due to chance or confounding by unmeasured variables, this association may also be due to poorly understood effects of the maternal gut flora on the immune system of mothers not undergoing labor, which may ultimately influence neonatal immunity.

Similar to previous studies, we found an independent association between maternal history of atopy and secretion of IL-13 [9-11] and IFN-γ [11,12] by CBMCs. This association was not related to maternal gut colonization or cesarean section delivery, as we did not find evidence that the maternal stool flora or mode of delivery differed between atopic and nonatopic mothers. Although the allergic status of the mother was not confirmed by measurement of serum IgE specific to allergens or by allergy skin testing, self-reported atopic diseases have been shown to be correlated with objective markers of atopy in mothers of children participating in similar studies [39]. The reason for frequently finding the strongest and/or most statistically significant associations between the exposures of interest (e.g., cesarean section) and particular patterns of cytokine secretion by CBMCs after stimulation with dust mite (*Der f 1*) and cat (*Fel d 1*) allergens but not after stimulation with mitogen or LPS is not clear. It is plausible that these two inhalant allergens are more widely distributed in the environment[40] and therefore maternal exposure to these allergens during pregnancy result in transplacental transfer of these allergens to the fetus leading to allergen-specific T cell priming in utero [41,42].

We recognize several additional limitations to our findings. First (as previously mentioned), the data has to be interpreted as preliminary because of small sample size resulting in limited statistical power and inability to adequately control for potential confounders. However, we found consistent associations between cesarean section and neonatal secretion of cytokines. Furthermore, we found consistent correlations between various bacterial species in maternal stool and specific cytokine patterns in neonatal cord blood that differed by mode of delivery. Second, we did not have adequate statistical power to test whether the pattern of cytokine secretion at birth is different in children born by elective versus non-elective cesarean section. Because a majority of the study participants who had a cesarean section had it scheduled, elective cesarean section is likely responsible for our findings. Third, although we did not select our cohort based on a family history of atopy, approximately 60% of the mother reported having a history of atopy which is higher than reported from the general population; therefore the results may not be generalizable to the general population. Fourth, we recognize that IL-10 is secreted by cells other than Tregs (e.g., Th2 cells). However, we found an inverse association between cesarean section and IL-13 (a Th2 cytokine) and differences in the association between specific microbes in maternal stool and secretion of IL-13 vs. IL-10 by CBMCs, suggesting that neonatal IL-10 was likely secreted by Tregs (a major source of this cytokine) [19-21,43]. Other studies have found increased secretion of IL-10 and IFN-γ by CBMCs in response to stimulation with LPS [44], peptidoglycan (a cell wall component of gram-positive bacteria) [45], and mycobacterial extract (PPD) [12]. Together with our findings, these results suggest that different microbial stimuli can impact specific subsets of T cells in the fetal immune system such as Th1 cells and T regulatory cells. Fifth, potential contamination with LPS may influence the interpretation of cytokine responses in cord blood. However, the endotoxin contents present in the allergens (*Der f 1 *and *Fel d 1 *) and mitogen (PHA) samples (≤ 1.23 EU/mg) were 1 × 10^6 ^times less than the concentration of LPS stimulus used in this study. Finally, maternal infection or antibiotic use around the time of birth was not an exclusion criterion in our study. However, the white blood cell (WBC) count of participating mothers at the time of delivery (median = 10 × 10^9 ^cells/L) was not significantly associated with secretion of any cytokine by CBMCs (P > 0.20 in all cases). In addition, there was no significant difference in WBC count between mothers who had a vaginal delivery and those who had a cesarean section (P = 0.82).

In conclusion, our findings suggest that cesarean section leads to abnormal neonatal immune responses (increased secretion of IL-13 and IFN-γ) that may precede the development of asthma and atopy. In addition, results of our exploratory analyses suggest that exposure to specific microbes (e.g., lactobacilli and bifidobacteria) in the maternal vaginal flora may influence neonatal immune responses in children born by vaginal delivery. These hypotheses will need to be examined in future longitudinal studies with large sample size.

## Abbreviations

T- helper 1 (Th1); T- helper 2 (Th2); Interferon-γ (IFN-γ); Interleukin (IL); cord blood mononuclear cells (CBMCs); lipopolysaccharide (LPS); phytohemagglutinin (PHA); *Dermatophagoides farinae 1 *(*Der f 1*); *Felinus domesticus *(*Fel d 1*); T regulatory cells (Tregs); capsular polysaccharide complex (CPC); capsular polysaccharide A (PSA); natural killer (NK) cells.

## Competing interests

Scott T. Weiss received a grant for $900,065, A**st**hma Policy **St**udy, from A**st**raZeneca from 1997 to 2003. He has been a coinve**st**igator on a grant from Boehringer Ingelheim to inve**st**igate a COPD natural hi**st**ory model that began in 2003. He has received no funds for his involvement in this project. He had been an advisor to the TENOR **St**udy for Genentech and has received $5,000 for 2003–2004. He received a grant from Glaxo-Wellcome for $500,000 for genomic equipment from 2000 to 2003. He was a consultant for Roche Pharmaceuticals in 2000 and received no financial remuneration for this consultancy. None of the other authors of this manuscript has any competing interests.

## Authors' contributions

N.P.L. conducted the data analysis and wrote the manuscript. B.R-P, A.O.T., and D.R.G. performed the cytokine measurements. A.O., M.L.D., and A.M.D. conducted all of the microbiologic studies. A.A.L., H.L., and L.R. assisted and supervised the data analysis.

C.L. and D.L. recruited the participating subjects. S.T.W. participated in study design and obtained funding. J.C.C. participated in study design, data analysis, and obtained funding.

All of the authors participated in drafting the manuscript and approved its final version.
